# Meeting October 4

**Published:** 1886-11

**Authors:** 


CHICAGO MEDICAL SOCIETY.

*Stated Meeting, October 4, 1886.*—E. J. DOERING, M.D., PRESIDENT, in the chair.

PROFESSOR CHARLES WARRINGTON EARLE entertained the Society with a very interesting account of his recent trip abroad, referring more particularly to observations made in the

HOSPITALS OF PARIS AND THE LABORATORY OF M. PASTEUR, an abstract of which appears elsewhere in this number of the JOURNAL AND EXAMINER.

DR. CHARLES F. SINCLAIR read a paper on

THE RELATION OF CERTAIN FORMS OF DEFECTIVE VISION TO HEADACHE IN YOUTH.

He said that the headaches arising from defective vision are very numerous. They possess certain definite characteristics, according to the degree and character of the ametropia. Thus, instead of the usual classification, the ophthalmologist might furnish a terminology of his own based upon the condition of the eye. The pain in these cases increases as the errors of refraction become more complicated. A woman had suffered with intensely painful headaches for fifteen years who was found to have mixed astigmatism, and here properly adjusted lenses effected a cure.

It is in childhood and youth especially that these different forms of ametropia manifest themselves in headache. Dr. W. H. Day, of London, however, in his work on the "Nature and Causes of Headache," in which he devotes a lengthy chapter to the headache of childhood and youth, does not mention ametropia as a possible cause.

Nevertheless the eye, among school children, is frequently the cause of all the head trouble. Among American children one form of ametropia is exceedingly common and very disastrous in its effects. It is difficult to detect. It may in certain cases simulate different forms of ametropia, and even normal vision. He referred to a case in which occurs a slight degree of astigmatism under one dioptric.

Maud W., a school girl 14 years old, had suffered with severe headaches. On examination excellent vision was found, but minus lenses improved it and made it normal. After the use of homatropin, however, half a dioptric of hypermetropic astigmatism was found, which was corrected and the headache cured.

Emily R., 15 years old, had suffered with constant headache for a year. On examination, .50 D manifest hypermetropia was found, and only this was corrected, as patient would not submit to the use of atropia. This patient returned in a few days saying that her headaches were as bad as ever, when homatropin was used and a small degree of hypermetropic astigmatism was found. This being corrected, a cure of the headache was effected.

In another case there were constantly recurring attacks of vertigo and dizziness, together with severe unilateral headache caused by astigmatism, and which properly adjusted cylindrical lenses cured.

These are types of an exceedingly large number of cases where headache can be cured by a weak cylindrical lens. These cases are interesting not only because of their marked consequences, but because of their tendency, in certain cases, to exactly simulate other conditions, and they certainly suggest the advisability, in every case of severe headache, to examine the eye only when under the influence of atropia.

PROFESSOR W. FRANKLIN COLEMAN said that in an examination of the eyes of pupils in Boston, in eighty-nine cases of defective vision fifty-nine were traceable to over-study. Out of this number twenty-five to fifty per centum of the cases of headache which occurred were relieved by supplying suitable glasses. Another class of cases in which headache occurs is when there is weakness of the recti muscles. By supplying proper prismatic glasses this weakness may be overcome, and the headache disappears. Other factors in producing headache are improper light and too low desks, thus causing the pupil to strain the eyes. So far as artificial light to read by is concerned, the incandescent electric light is probably the best, as it most nearly resembles sunlight, and is free from heat, dust and flickering.

PROFESSOR F. C. HOTZ said he believed the authors of such papers as this often exaggerate the importance of their observations. The result is, patients are often advised to go to oculists, but do not do so on account of the expense, or for some other reason, and the patient therefore receives no treatment. But he has been surprised to see how few cases of defective vision come to him complaining of headache. He has not found it a prominent symptom. In looking over his record of cases he finds a great many cases of astigmatism, hypermetropia, and ametropia, in which the symptoms complained of are difficulty of vision, but not headache. Children often complain of not being able to see the figures on the blackboard, and consult the oculist for that trouble directly. But if a large number of cases of the eyes of children are examined, it will be found that in youth a normal eye is often far-sighted, and yet such persons are able to do the work required. However, in cases of defective vision, accompanied by headache, the defect of vision should be corrected.

PROFESSOR J. E. COLBURN related his personal experience with astigmatism, stating that not only had he headaches, but he could not study well, and these difficulties disappeared with the using of proper glasses.

